# Remote Eye Triage: Health Economic Perspectives on Resource Prioritization

**DOI:** 10.1177/11786329251347684

**Published:** 2025-06-26

**Authors:** Casper van der Zee, Robert P. L. Wisse, Saskia M. Imhof, Miriam P. van der Meulen

**Affiliations:** 1 Department of Ophthalmology, University Medical Center Utrecht, The Netherlands; 2 Department of Epidemiology and Health Economics, Julius Centrum, University Medical Center Utrecht, The Netherlands; 3 Erasmus School of Health Policy and Management (ESHPM), Erasmus University Rotterdam, The Netherlands; 4 Xpert Clinics Oogzorg, Zeist, The Netherlands

**Keywords:** health economic methods, complex interventions, remote eyecare, telemedicine, delayed care, LOGIC model

## Abstract

**Background:**

The TeleTriageTeam (TTT) is a novel system for remote eyecare delivery.

**Objectives:**

Explores the impact of TTT on costs by depicting program theory of care prioritization. Moreover, a quantification of longer-term effects of delay (and inversely earlier treatment) on costs is performed.

**Design:**

Mixed-methods health economic evaluation.

**Methods:**

First, we depicted the program theory of prioritization into a LOGIC-model using existing TTT-data and expert interviews outlining the causal pathways how and why a program is expected to work. Second, we identified the most relevant key diagnoses to be appraised based on incidence, severity, and presumed triage impact. Third, we estimated the impact of delay (or inversely earlier treatment) on societal costs and quality of life (QoL) based on literature searches. Cost data were updated to 2023. Results were reported per delayed patient per 6 months (the average TTT delay).

**Results:**

Five key diagnoses were selected: cataract, diabetic retinopathy (DRP), age-related macular disease (AMD), glaucoma, and dry-eye-syndrome (DES). The LOGIC-model showed how the TTT actions could influence costs and QoL. Semi-structured interviews revealed delay results in adverse events, at the expense of shorter waiting times in prioritized patients, and overall decreases personnel burden. Reduced waiting times were also believed to decrease burden and costs in prioritized patients. Literature showed that a delay in glaucoma treatment results in savings (−€409), while the other diagnoses suggested higher societal costs (cataract €3298, DES €2156, AMD €1455, DRP €117). QoL reduction and increased costs due to delay were more apparent when delay results in longer duration of curable symptoms compared to delay in stable disease (up to 0.09 vs 0.003 QALYs and €3298 vs €1455, respectively).

**Conclusions:**

Eye care delay results reduced QoL and increased societal costs, yet this is compounded by gains attributable to justified prioritization of more urgent and more severe patients.

## Introduction

Access to care is stressed, particularly in eye care, driven by factors such as technical possibilities, patient expectations, longevity and an overstretched workforce.^1^ The number of patients is growing twice as fast the workforce of trained practitioners.^2^ Moreover, learnings from the COVID pandemic highlighted the consequences of a sudden stop to care; our institutions’ outpatient eyeclinic reduced it’s activity by 90%, disrupting the continuity of over 300.000 annual patient interactions yearly. A report of the Dutch *National Institute for Public health and the Environment* on the consequences of delaying surgeries during the COVID-19 pandemic estimated ophthalmology being responsible for 38% of all QALY lost in health care, mostly due to interventions not being performed.^3^ This is due to high volumes in ophthalmic care combined with visual acuity reduction being impactful on the quality of life, and underlines that ophthalmology is a relevant field to assess the access to care.

**Figure 1.**
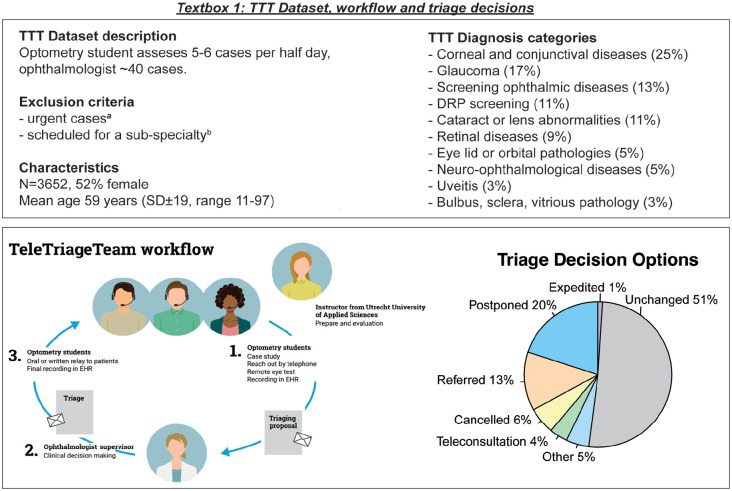
TTT dataset, workflow and triage decisions. Demographics, clinical characteristics, and triage outcomes of patients assessed by the TeleTriageTeam. ^a^For example, neovascular age-related macular disease, poorly regulated glaucoma, uveitis, retinal detachment, or keratitis. ^b^Patients waiting for their uveitis-, pediatric-, or vitreoretinal consultants. Two cornea consultant participated in TTT, enabling triaging of subspecialty cornea cases.

In this environment, the TeleTriageTeam (TTT) was conceptualized in spring 2020 as a means to utilize limited resources for the most urgent and severe cases, and to double down on delivering remote care for continuity. The TTT works with a team of optometry-students whom contacted patients waiting for a planned hospital consultation by phone. The students received direct supervision and coaching by their optometrist-teachers from the University of Applied Sciences Utrecht. After summarizing the medical record, the patients’ response, and the current health status, the students proposed a triaging strategy (eg, schedule, postpone, cancel, or expedite care). These triaging proposals were judged by an ophthalmologist in a plenary clinical case conference. Subsequently, the students relayed the triage decision to the patient and the outpatient clinic. The TTT managed to redirect half of all appointments to an alternative of a physical consult (see Figure 1). This means the consultation was either conducted by telephone, delayed, referred from academic to a regional physician, or not deemed necessary.^4^ On the short term, TTT therefore reduced care utilization in not so urgent cases, effectively creating space for more urgent or severe cases.

Yet, these increased efficiency gains and short-term cost-savings could potentially be made at the expense of the quality of life of the rejected, referred or rescheduled patients.^3^ To understand the long term value of TTTs prioritizing (by remotely triaging and restructuring scheduled appointments and restructure the soaring waiting lists), this should be analyzed in a health economic evaluation (HEE). A HEE provides a systematic way to assess the value of interventions, helping policymakers to allocate resources efficiently. Over the years, TTT proved to be of considerable value to the hospital, the patients, and the optometry school. Yet the effectiveness in terms of health effects, as well as the economic value of this complex experiment is difficult to quantify, since TTT was conceptualized under great stress without prospective clinical data collection, consent for follow-up research, or a potential control group. Therefore, barriers exist to formally perform a cost-effectiveness analysis. As described in the framework for complex interventions and natural experiments, information on the program theory and a quantification of expected (health-economic) outcomes is essential as a first step to inform professionals, eye care institutions, and policy makers. Here, program theory refers to a structured explanation of how and why a program is expected to work, outlining the causal pathways between activities, mechanisms, and intended outcomes. In this paper, we conceptualize how and why the TeleTriageTeam impacts healthcare utilization, costs, and quality of life.

## Methods

### The Analysis in Summary

The TTT was designed and deployed as a clinical service, not necessarily suited for evaluating of clinical effectiveness and (economic) quantification. Therefore, it lacks a direct comparison group. To gain insights in the (economical) value of this complex intervention, we combined three methodological steps in line with two existing frameworks.^5,6^ First, we mapped the program theory based on the curated data during the TTT project, and on semi-structural interviews with clinical experts to understand the impact of triaging decisions. Second, we determined the most relevant eye diagnosis to be appraised, based on incidence, severity, and presumed triage impact, hereafter referred to as “key diagnoses.” Third, we explored the impact of delayed care (and inversely earlier treatment) on costs and QALYs based on literature data.

### The TeleTriageTeam Design and Work Flow

The TTT is an innovative method to deliver remote eyecare, using a web-based eye test, teleconsultations, and advanced task redistribution. Its purpose was to help prioritize scheduled appointments and restructure the (soaring) waiting lists. The project and its clinical outcomes were reported in depth previously.^4,7^ In summary, the TTT consists of optometry students, a tutor optometrist, and a supervising ophthalmologist. The students received a 2-day training program focusing on navigating the electronic health record, clinical best practices, and patient communication. TTT focused on summarizing the patients clinical history, after which they contact patients scheduled for an elective outpatient follow-up consultation under supervision of a qualified tutor optometrist (see a patient flowchart in Figure 1). The student evaluated a patients health status by conducting semi structured anamneses by phone. If visual acuity was of interest by the TTT, patients were requested to perform a remote, self-administered online test at home. Patients were called back after their cases had been discussed by the supervising ophthalmologists in a clinical case conference. A decision was made either to continue schedule the appointment, or to provide an alternative (postpone, refer, cancel, remote or expedite).

### The TeleTriageTeam Acquired Data

For this study we used the same consecutive 3658 cases from April 16 2020 to December 31 2021 as in the previous study so that the results and conclusions can be compared. The TTT excluded highly urgent cases (eg, neovascular age-related macular disease, poorly regulated glaucoma, uveitis, retinal detachment, or keratitis) and patients scheduled for a sub-specialty (eg, patients waiting for their uveitis-, pediatric-, or vitreoretinal consultants). The TTT offered multiple triaging decisions. In 49% of cases, an alternative to the physical consult was provided due to the TTT (Figure 1). The analysis focusses on the 20% patients that were delayed (n = 733, on average by 6 ± 4 months). Other triage decisions were referral (13%), cancelled (6%), teleconsultation (4%), expedited (1%), others (5%), and unchanged consultations (51%). This paper reports on the effect of postponement, as it was the most common triage decision, although patients referred to another center or cancelles probably also had a delay in their care pathway. Importantly, delaying patients creates potential gains for other prioritized patients. These trade-offs are illustrated in the analysis. A sample size calculation was not applicable due to the descriptive nature of this dataset. All patients of this data set were included for analysis.

### Step 1: Mapping Program Theory and Their Influencing Factors

The program theory, containing the intended effects and outcomes of TTT, was visualized in a LOGIC model. This methodological step was based on three frameworks: (1) the purposeful program theory, (2) the framework for complex interventions, and (3) the framework for natural experiments.

#### Purposeful Program Theory and the LOGIC Model

**Figure 2.**
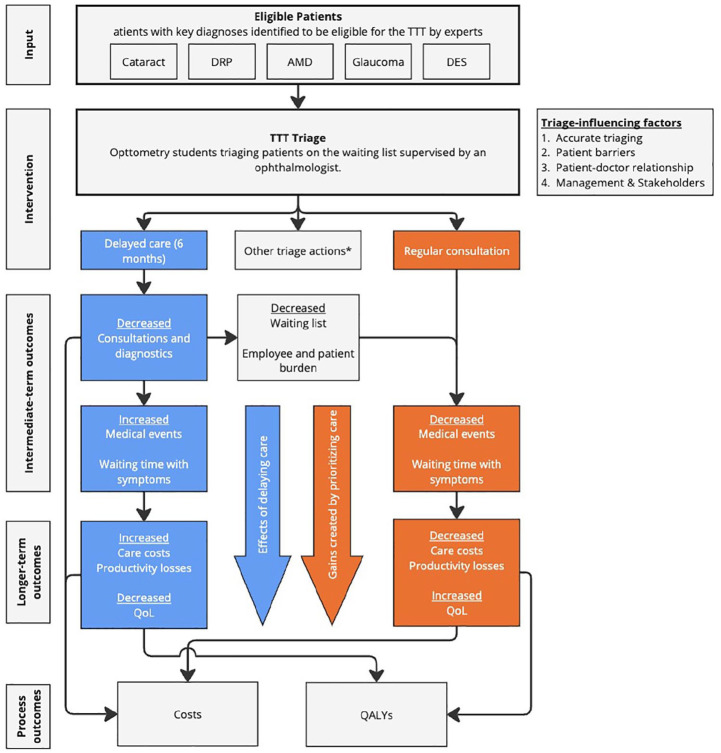
LOGIC model on program theory of the TeletriageTeam (TTT), as per the conceptual framework by Funnell and Rogers. Abbreviations: AMD, age-related macular disease; DES, dry eye syndrome; DRP, diabetic retinopathy; QALY, quality-adjusted life years; QoL, quality of life. The effect of the TTT is displayed by the negative effects of delay (blue pathway) versus the potential gains created by delaying others (orange pathway). *Other actions than delay or regular consultation are reported in Figure 1.

To gain insight in the outcomes of TTT as a complex intervention, we constructed a LOGIC model, based on *purposeful program theory* by Funnell and Rogers.^8^ Usually, a study solely assess the outcomes of an intervention. However, how and why the intervention does(n’t) work is not assessed. This is referred to as a black box. The purposeful program theory explains how and why an intervention is expected to achieve its goals by outlining the (causal) connections between inputs, activities, outputs, and outcomes, which can be visualized using a LOGIC model.^8^ It includes underlying assumptions and external factors that influence success, providing a rationale for the program’s design. Within this context, we developed a LOGIC model (Figure 2).

#### Two Frameworks: Complex Interventions and Natural Experiment

Two frameworks were used as a rationale to map potential outcomes in the LOGIC-model: “*complex interventions*” and “*natural experiments*” (NE).^5,6^ The complex intervention framework^5^ describes core elements to assess and develop interventions. An overview of the core elements used for this analysis is found in Supplementary File 2. Second, the TTT is considered a natural experiment^6^ as “naturally occurring” means not being developed from a research context, contrary to Randomized Controlled Trials (RCTs). Given that RCT designs are usually not suited to evaluate NEs, Deidda and colleagues inform the difference between reporting guidelines of RCTs vs NEs, transparent and correct usage of observational administrative data, reporting of data, and the usage of secondary sources for the assessment of effectiveness and costs (eg, literature searches and expert consultations used in this paper). Therefore, their checklist served as a reporting guideline.

#### Semi-Structured Interviews to Map Outcomes and Identified Key Diagnoses

Semi-structured interviews were used to map the LOGIC model with program theory by evaluating relevant outcomes of TTT (step 1) and identifying key diagnoses (step 2). This approach where the LOGIC model is informed trough literature searches, author team discussions, and interviews with experts is suggested by the European *Guidance on the use of Health Technology Assessments LOGIC models in complex interventions.*^9^ For the interviews, a topic list was developed delineating themes and corresponding questions designed to identify the effects and outcomes of delayed care (Supplementary File 1). The topic list questions were not derived from a validated questionnaire, as they were tailored to address specific key topics of interest related to TTT. During the semi-structured interviews, five experts were selected for interview from a purposive sampling approach. These experts were eye care professionals as the research team selected them to be key opinion leaders in the field. Characteristics of the interviewees are reported in Supplementary File 5. The experts varied in setting (urban/rural), hospital (academic/community/ambulatory), expertise in telemedicine (yes/no), and expertise in supervising the TTT (yes/no). Before the interview, experts were informed on the background of TTT and the interview’s objective through an email, as well as a presentation given immediately before the interview. The primary topics during the interview were (1) which (groups of) patients would be most or least suitable for TTT assessment, and (2) what the expert considered relevant effects and outcomes of TTT. Example questions were: “who is in your opinion the typical patient best helped by the TTT?,” “in what situations would you expect the TTT to reduce workload?” or “which patients are at risk for poor remote triaging by TTT?.” Interviews were anonymously recorded, edited transcription was applied, and transcripts were analyzed thematically. A codebook and topic list were developed in advance of transcription based on the topic lists. Coding insights emerged during transcription were added. Interviews were enriched with the experiences of the research team, exists of two ophthalmologists (SI and RW) experienced in supervising the TTT, and two medical doctors with expertise in health economic evaluations (MM and CZ).

### Step 2: Selecting the Appropriate Key Diagnoses

Health-economic analyses typically evaluate interventions in a specific target population. Yet, the population of TTT is heterogeneous. Therefore, we quantified outcomes by selecting key diagnoses in a two-stage process. The first selection was based on occurrence in the TTT dataset, the severity of the disease, and the impact of triaging (eg, whether a care pathway was expected to be suited for TTT). Then, the interviewed naïve experts with and without TTT experience were asked which diagnoses they thought would be (not) eligible for TTT triaging and subsequently whether they agreed with the research teams’ proposal (see Supplementary File 1 for the interview topic list).

### Step 3: Literature Search on the Identified Outcomes: Costs and QALYs

Costs and quality adjusted life years (QALYs) were estimated that could result from delay or postponement, as it was the most common triage decision, while patients referred to another center or cancelles probably also had a delay in their care pathway. Inversely, earlier treatment because of prioritization would have an opposite effect. The literature was searched for utilities, quality adjusted life years (QALYs), healthcare resource use, missed events due to delay of care, and costs. The search was performed in Medline and Embase according to Paisley’s minimum search requirements^10^ and search input from suggestions in the literature.^11-13^ Costs are analyzed from a societal perspective, which includes direct medical costs (eg, consultations), direct non-medical costs (eg, travel expenses, informal care), and indirect costs related to productivity losses. Informal care refers to unpaid assistance provided by family members or other non-professional caregivers, often involving help with daily activities or support during illness. Keywords and search criteria are found in Supplementary File 4. Costs were updated using the Consumer Price Index and the *Hospital Services* Basket of the Purchasing Power Parities to recalculate to the most recent dataset of 2023 Euros using the medical basket approach.^14-16^

## Results

### Step 1: Mapping Outcomes and Influencing Factors

When interviewing the expectations on the impact of TTT on costs, experts primarily referred to reduced short-term, in-hospital costs, such as avoided consultations and diagnostics, not taking into account a poorer health state due to waiting with curable symptoms or the consequences of missed events. However, when asked about the prioritized or urgent patients, experts indicated they expect that eyecare primarily aims to improve clinical outcomes, and thereby a patients’ quality of life (QoL). Inversely, treatment delay results in a decrease in consultations and diagnostics. Experts considered that a rise in medical adverse events and increased waiting time with symptoms was unavoidable with postponement, particularly in unstable chronic conditions. This was expected to result in increased costs, productivity losses in patients, and QoL decline. These effects were illustrated as the blue pathway in Figure 2. The experts highlighted that a focus on stable conditions also eligible patients were believed to minimize these effects.

The TTT is expected by experts to reduce the burden on both patients and staff due to a decrease in planned consultations, reducing travel costs and time spent in waiting rooms for the patient. Staff burden might be reduced because they see less patients and spend time on more urgent and severe cases. Yet, experts also expected an increased complexity of patients over time, given the challenging patients are selected to remain in the academia. Experts expected that the effects of delay would results in gains for the prioritized patients due tie less shorter waiting times, thereby resulting in less medical events, and thereby costs and QoL (orange pathway). Therefore, the relevant outcomes of TTT identified during the experts-interviews were: costs, quality of life, burden on patients and staff, waiting times, and medical events.

#### Influencing Factors by Expert Opinion

All interviewees stressed that essential factors need to be fulfilled to accomplish safe postponement. To this end, four influencing factors were identified: accurate triaging, patient barriers, patient-doctor relationship, and stakeholders management. First, accurate triaging, that is, the ability to correctly distinguish patients for safe postponement, was considered the most important factor. Second, experts identified patient barriers that could impact adherence to triaging advises, for example, contacting the clinic by phone for emergency consultations, seeking parallel redundant care, that is, second opinions. Therefore, some patients were expected to benefit less from TTT, such as patients from a cultural background with structurally different health systems, or with poor language proficiency. Third, trust and a good patient-doctor/hospital relationship influences patient adherence to the TTT advises, which is believed to impact the adherence of the TTT advise to patients. The last identified influencing factor is the management and stakeholders (eg, IT, hospital governance, and overhead). Experts mentioned “*this is easily overlooked, but each stakeholder is dependent on the other, and if one does not commit, the team cannot function properly*.”

### Step 2: Identification of Key Diagnoses

Experts were unanimous in which diagnoses they expectated to be eligible for TTT. They mentioned that because of the potential harm caused by delay, cases could only be delayed when certain disease characteristics were met, such as stable disease or stable retinal imaging scans. Moreover, experts believe there is a difference between patients with continuous symptoms being delayed and patients with stable disease. In the first, delay has impact on health and resource use due to prolonged symptoms (eg, cataract). The latter, stable disease, represent stable or chronic conditions on which delay does not directly affect quality of life, but induces a probability of an adverse event (eg, glaucoma). Experts expected TTT will have a higher beneficial impact on populations with the first diagnoses compared to the latter.

The key diagnoses included in the analysis are cataract, Dry Eye Syndrome (DES), Diabetic Retinopathy (DRP), Age-Related Macular Disease (AMD), and glaucoma. All experts agreed cataract and DES to be representative diagnoses for the analyses due the high occurrences. DRP, AMD, and glaucoma were identified as diagnoses with high occurrences (and therefore have a high demand on care personnel), and eligible for remote clinical advise to patients. AMD, specifically, is usually treated with repeated retinal scans combined with expensive intra-vitreal injections every 4 to 12 weeks (IVI). Though physical consultations are needed for these scans and injections, experts indicated that those who exhibit stable disease, would have relative minimal harm from delay. Experts suggested patients with the proliferative version of DRP are likely to experience harm by delay, and were therefore not postponed. In contrast, experts did not expect previously stable DRP or glaucoma to be at increased risk due to a treatment delay (eg, diabetic CNV or acute glaucoma). Experts also mentioned non-urgent diagnoses that would likely be harmed by delay (and inversely benefit from shorter waiting time), such as uveitis, progressive keratoconus, and multicomorbidity).

### Step 3: Literature Search on Costs and Effects

To provide insight in the potential impact of delay of care delivery across key diagnosis, costs and quality of life data were derived from literature. Details of the literature assumptions is described in Supplementary Table 1. The impact of delay on the various cost-components were based on logical assumptions mentioned in Supplementary Data File 3. For instance, surgery costs of cataract surgery were not considered as cost-savings as they would presumably still occur 6 months later and were thus not avoided. The likelihood of adverse missed events were estimated based on literature and recommended national guidelines. The most relevant assumptions are:

– *Cataract care*: surgery costs were not included since it is assumed that surgery is delayed rather than avoided.
– *Age-related macular disease (AMD) and diabetic retinopathy (DRP)*: delay is expected to reduce the consultation- and medication-direct medical costs, yet also to induce missed events. DRP: only patients without CNV and DME.
– *Glaucoma and dry eye syndrome (DES)*: patients were assumed not to have additional medical interventions (eg, surgery) since only stable patients are postponed in the triaging process.

#### Short-Term Direct Medical Costs

Table 1 reports the variables used in the analysis, relevant background information is provided in Supplementary Table 1. Average costs due to 6 months delay are reported in Table 2 and lead to up to −€409 medical savings per case during the half year. This number did not take into account the costs of any missed events or parallel (external) healthcare consumption. A detailed explanation on the assumptions used for these calculations is described in Supplementary File 3.

**Table 1.** Reported values informed by literature in 6 months.

Key diagnoses	Medical costs^†^ (€)	Δ Utility^†^	Working pop. (%)	Occurrence missed event (%)
Dry eye syndrome	424^17^	0.05^18^	59.0^19^	0*
Cataract	0^d^ [EO]	0.07-0.09*,^20,21^	10.6^22^	0*
Age-related macular disease^a^	581; 663^b^,*,^23^	0.095^24^	3.37^22^	59^25^
Glaucoma^a^	409^26^	0.10^27^	20.7^22^	0.0031^28^
Diabetic retinopathy^a^	86^29^	0.075^30^	50.0^31^	3.7^32^
*Other costing variables*				
*Parameters*			*Values*	
Out-patient consultation			€120^33^	
Intra-vitreal injection			€286^34^	
OCT scan			€62 [RR]	
Laser PI			€379 [RR]	
Glaucoma medication			€31 [RR]	
Event: CNV, ME			Consultation + OCT + IVI [EO]	
Event: acute glaucoma			Consultation + OCT scan + PI laser + medication [EO]	
Productivity costs: VA loss			20.6% less^35^	
Average workweek			32.1 hours^b,33,36^	
Wage			€39.88/hour^33,36^	
Employed labor force			70.4%^c,33,36,37^	
Informal care use			5.8 hour per week^38^	

Abbreviations: CNV, choroidal neovascularization; EO, expert opinion; IVI, intra-vitreal injection; ME, macular edema; OCT, optical coherence tomography; OECD, Organization for Economic Cooperation and Development; PI, peripheral iridotomy; RR, reimbursement rate; UMCU, University Medical Center Utrecht.

Costing variables and utility used for the analysis, informed by literature, per diagnose. Prices are in Dutch 2023 Euro (recalculated tot 2023 using the World Bank CPI and hospital PPP basket of the country of origin and the hospital PPP basket of The Netherlands based on OECD data).

See Supplementary Data File 3.

[] = Source.

a Stable disease.

b Full time is 36 hours per week for the average population, this is weighted for part time workers as well. Assumed is 47 working weeks per year.

c 15 to 75 years old.

d Cataract direct medical costs are not included since it is assumed by expert opinion that the surgery takes place anyway with or without the TTT, though a few months later.

* Upper and lower bound.

Columns marked with the “†” symbol refer to the variable per patient per 6 months, the average delay of TTT.

**Table 2.** Average calculated differences per patient per 6 months delay.

Diagnoses	QoL difference (QALY)^†^	Medical cost (€)^†^	Productivity costs (€)^†^	Informal care costs (€)^†^	Total societal costs (€)^†^
Cataract	0.07-0.09^b^	0	463	2835	3298
DES	0.05	−424	2580	0	2156
AMD^a^	0.056	−581; −663^b^	87	1673	1373;1455^b^
DRP^a^	0.003	−86	81	105	117
Glaucoma^a^	0.00031	−409	0	0	−409

Abbreviations: AMD, age-related macular disease; DES, dry eye syndrome; DRP, diabetic retinopathy; QALY, quality-adjusted life years; QoL, quality of life.

Societal cost differences if a patient would be delayed by 6 months. Costs are in 2023 euros (€), negative costs are savings.

For all assumptions, see Supplementary File 3.

a Stable disease.

b Upper and lower bound.

Columns marked with the “†” symbol refer to the variable per patient per 6 months, the average delay of TTT. Literature for input variables are referenced to in Table 1.

#### Productivity Losses and Informal Care

Productivity losses and informal care data per diagnoses were not available in literature. Therefore, we calculated these costs with their relationship with vision loss as described in recent European studies.^35,38^ Details in calculation are described in Tables 3 and 4.

**Table 3.** Calculations and variables used for medical costs and societal costs: cost calculations.

Diagnoses	Medical costs	% in working population	Missed events	Average costs medical missed event	Include societal costs	Average productivity costs pp	Average informal care costs pp	Som direct costs^a^	Som societal costs^a^ for delay
Cataract	€0	10.60%	0	€−	Fully	€463.38	€2835.04	€−	€3298.42
AMD upper limit	€−581.32	3.37%	59%	€276.66	Only missed events	€86.92	€1672.67	€−304.65	€1454.94
AMD lower limit	€−663.09	3.37%	59%	€276.66	Only missed events	€86.92	€1672.67	€−386.42	€1373.17
DRP	€−85.84	50%	4%	€17.35	None	€80.87	€104.90	€−68.49	€117.28
Glaucoma	€−408.78	20.70%	0%	€0.02	none	€0.03	€0.09	€−408.77	€−408.65
DES	€−423.91	59.01%	0%	€−	Only productivity included	€2579.65	€−	€−423.91	€2155.74

a Negative values are savings.

**Table 4.** Calculations and variables used for medical costs and societal costs: variables used for cost calculations.

Variables	Values
Wage hour (€)	39.88
Average workweek (n)	32.2
Workweeks per half year (weeks)	23.5
Productivity loss (%)	20.58
Employed labor force (%)	70.39
Informal care (€ hour rate)	18.80
Informal care (hours)	5.8
Cost OCT (€)	62.31
Outpatient consultation (€)	120.00
Anti-VGEF injection (€)	286.62
Glaucoma medication (€)	31.36
PI (€)	379.04

Direct medical cost savings are overall outweighted by societal costs, except for glaucoma as the delayed glaucoma patient is not expected to be different compared to the non-delayed glaucoma patient in terms of societal costs (Table 2, Supplementary File 3). Delay is expected to result in the highest societal costs for DES and cataract; up to €2156 and €3298 per patient. AMD is calculated to result in an increase between €1373 and 1455, driven by the frequent occurrence of missed events.^25^ Costs for DRP are lower compared to the other diagnoses (117), and glaucoma reports a costs decrease of −€409, both due to low expected missed events in the case of delay.

#### Quality of Life Losses

Impact on quality of life in health economic evaluation is displayed in utilities, which range between 0 (corresponding to death) to 1 (perfect health). A QALY loss of 0.1 corresponds to ± half a month in perfect health). Table 2 reports the effect of delay on QoL, according to the literature. Delay is suggested to have the highest impact on cataract, DES, and AMD (0.05-0.09), and a limited impact DRP and glaucoma (⩽0.003).

## Discussion

This paper explored the potential impact of early remote assessment in eye triage by mapping the program theory, and by assessing the potential impact of delay on QALYs and costs. Three relevant findings emerged. We remark that TTT had multiple outcome decisions (eg, cancellation, referral, remote care) and this paper focusses on the effects of the postponement of health care delivery. First, we identified five effects of TTT on care through interviewing experts: costs, quality of life, burden on patients and staff, waiting times, and medical events. The program theory hypothesized the impact prioritization of health care delivery describes by expert interviews (more urgent or severe patients are utilizing clinical resources, and these resources could be made more available by postponing less urgent and less severe cases). Second, we identified five key diagnoses (ie, cataract, glaucoma, DES, DRP and AMD) of which four were deemed eligible for postponement of care. AMD was found to have an unacceptable high rate of missed events (59%) when delayed due to the delay in treatment of intra-vitreal injections (Table 1). Third, we estimated the potential harm that the delay of health care delivery could result in using existing literature reports as input. Quality of life reduction and increased costs due to delay were both more apparent when delay results in longer duration of curable symptoms compared to stable disease (up to 0.09 vs 0.003 QALYs and up to €3298 vs €1455, respectively). Costs were driven by societal components such as productivity losses and informal care costs, outweighing the short-term medical savings of delay (up to €3298 costs compared to up to −€409 savings respectively). These results regard the plain effects of the postponement. However, these effects are a trade-off with the potential gains of allocating the freed capacity to prioritized patients more in need. Without prioritization, random yet on average more severe patients are delayed.

Triaging solely on the basis of diagnosis is not sufficiently insightful to reveal the full effects of TTT on care. Experts clearly describe eligible patients in the form of key diagnoses, but they fail to outline a more comprehensive account of which patient is to be delayed where. In practice physicians seem to consider other variables than previously described, such as a patients’ visual acuity which highly impacts their utility difference.^30^ Though differentiation in diagnoses is important for non-triaging purposes, for example, for scientific comparison, these results show that the within-diagnosis variables are essential to be identified in future.

The clinical quality of correct triaging – being able to distinguish between higher and lower need for a consultation – is essential for such an intervention in order to be (cost)effective. For example, if many medical events are missed due to delay advised by the TTT in a few patients, at the small benefit of a few days earlier treatment in more patients, the intervention could controversially end up doing more harm than expected. Therefore, it is essential to assess the quality and accuracy of clinical triaging, which is ideally compared to a control group, via pre-post comparisons, or via patient matching. Since this intervention was designed as a clinical service during COVID, this comparison was not feasible and should be included in future research.

### Limitations

There are several limitations to consider. First, this analysis concentrates solely on the aspect of 6 months of delay, neglecting other outcomes of the TTT on care, such as cancelling, referral, or remote care delivery, and longer term outcomes than 6 months. This might result in selection bias. Second, we developed a LOGIC model informed by expert interviews. Though we focused on key diagnoses for the analysis, it has benefit to additionally validate the LOGIC model afterwards. Future research should include this validation step. Third, the questions of the semi-structured interviews could have been pilot-tested to increase the reliability of the results. Last, this study could be prone to confirmation- and selection bias. Confirmation bias should be considered since thematic analysis is subjective and relies on the researchers’ judgement, even though we explicitly asked interviewees for improvements and pitfalls. Selection bias as well since expert accepting invitations for interviews might have a preference for selecting certain outcomes or key diagnoses.

### Implications

This paper showed that a standard cost-effectiveness analyses is not feasible since the TTT was designed and deployed as a clinical service, not necessarily suited for prospective data collection during COVID, making pre-post or matching comparisons unfeasible as well. In addition, standard health economic evaluations generally have straightforward outcomes enabling early modeling exercises (eg, overall survival and progression free survival in cancer diagnoses). Even though our methods has been described in two frameworks and an EU guidance report,^5,6,9^ program theory and LOGIC models are not included in many papers yet. This paper is a showcase highlighting the value of this early assessment in practice as the LOGIC model informs on the intervention process, (intermediate and long term) outcomes, influencing factors, and relevant research questions. This provides guidance in methodology and data collection, for example relevant patient pathways, care consumption, patient delay, seeking care in other care facilities, follow-up, clinical outcomes, medical events, societal cost, and QoL. Moreover, all this data is ideally to be compared with a control group, pre-post comparison, or via patient matching. For researchers, this study underscores the value of embedding program theory and LOGIC models early in complex interventions to improve the quality and focus of data collection and evaluation strategies. For policymakers, it highlights how structured early assessments can inform funding decisions, support service design, and identify key outcome indicators relevant for monitoring impact over time. For the public, it shows that transparent modeling of healthcare interventions enhances accountability and helps ensure that new services are aligned with patient needs and societal benefit.

### Conclusion

In conclusion, the results showed that delay results in reduced quality of life and increased societal costs. However, these effects are a trade-off with the potential gains of allocating the freed capacity to prioritized patients more in need. Without prioritization, random yet on average more severe patients are delayed. Therefore, this effect is compounded by gains attributable to prioritization of more urgent and more severe patients. Overall, the LOGIC model identifies group benefits in prioritization of care delivery: more urgent and more severe patients are now utilizing clinical resources, and these resources are made more available by postponing less urgent and less severe cases. This early economic evaluation informs future research on the effects and costs of remote eye care. We underscore the need for a health economic evaluation that prospectively assess all identified outcomes and triaging accuracy.

## Supplemental Material

sj-docx-1-his-10.1177_11786329251347684 – Supplemental material for Remote Eye Triage: Health Economic Perspectives on Resource Prioritization Supplemental material, sj-docx-1-his-10.1177_11786329251347684 for Remote Eye Triage: Health Economic Perspectives on Resource Prioritization by Casper van der Zee, Robert P. L. Wisse, Saskia M. Imhof and Miriam P. van der Meulen in Health Services Insights

sj-docx-2-his-10.1177_11786329251347684 – Supplemental material for Remote Eye Triage: Health Economic Perspectives on Resource Prioritization Supplemental material, sj-docx-2-his-10.1177_11786329251347684 for Remote Eye Triage: Health Economic Perspectives on Resource Prioritization by Casper van der Zee, Robert P. L. Wisse, Saskia M. Imhof and Miriam P. van der Meulen in Health Services Insights
