# Supplementary material for: Remote Eye Triage: Health Economic Perspectives on Resource Prioritization
Source: Health Serv Insights. 2025 Jun 26;18:11786329251347684. doi: 10.1177/11786329251347684 (PMC12202912; doi:10.1177/11786329251347684)
Supplement: sj-docx-1-his-10.1177_11786329251347684 – Supplemental material for Remote Eye Triage: Health Economic Perspectives on Resource Prioritization [file sj-docx-1-his-10.1177_11786329251347684.docx]

**Supplementary file 1: Topic List Interviews**

**Goal questions of interview**

- Did we select the correct diagnoses?
- which diagnosis are important to assess remotely?
  - Why?
  - Would you advise additional diagnoses we did not include in the search?
- What are patients expected to do when they received TTT advise to delay care by six months?
  - will the patient switch health provider?
  - Wat are the expected consequences for the patient of earlier or delayed treatment?
  - What do you think will be the effect on Quality of Life on patients after 6 months delay?
  - What do you think will be the effect on health care costs if patients are 6 months delayed?

**Relevant information was send a few days before the interview by email. Expected duration of interview: 30-45 minutes.**

| **Introductie** |
| --- |
| Introduction of the interviewer. Brief background and purpose of interview. |
| *I am making an audio recording of this interview so that I can listen back and elaborate on this interview afterwards. The audio recording will not be shared with anyone and deleted after the interview is worked out. Is this okay with you?* |
| Do you have any questions before we move on to the content part of the interview? |
| Introduction expert: Can you briefly elaborate on your position as an ophthalmologist? Which patient populations in particular do you work with in your daily work? Does your work involve digital care for patients? |
| **Uitleg TeleTriageTeam (TTT)** |
| You have received information about the TeleTriage Team prior to this interview.   1. What was your first impression of the TTT?    - Could you further elaborate on this? 2. How would this help you as a caregiver in day-to-day practice?    - And how would this help your patients? 3. Could digital screening also negatively affect your work (environment)? |
| **Verwachte invloed TTT** |
| 1. What impact do you expect the TeleTriageTeam to have on patient care?    1. *Eg: Do you think the TTT will benefit health care?*    2. *Do you think patients are better off with TTT?* 2. What patients or diagnoses could potentially suffer (severe) harm from/due to TTT? 3. Do you see patients or diagnoses that could potentially benefit from TTT? Can you elaborate on this? 4. Do you see differences for different diagnoses/are there typical diagnoses that have advantages or disadvantages? 5. In what situation would you expect the TTT to reduce workload for employees? Or: how will the TTT be most effective? 6. If you wanted to explore the potential impact of teletriage, which groups of patients and diagnoses do you think would be most relevant? 7. Why did you choose these groups? *If not mentioned, ask specifically about cataract, DRP, AMD, glaucoma, dry eye syndrome* 8. Which characteristics first come to your mind? 9. Who is the typical patient who has been best served by the TTT? 10. Who is the typical patient that is at risk due to the TTT? 11. Who is the typical patient you expect to encounter most often at TTT? |
| **Follow-up of TTT advice after triage to patients** Our database shows that if an appointment is rescheduled, patients are seen an average of 6 months later. |
| 1. Suppose the patient is doing well and you advise to reschedule the appointment for 6 months. Do you expect the patient to agree to the telephone advice? 2. Do you see any impeding factors for patients to follow-up on this advice? If so, which ones? 3. Do you think patients will be harmed by the 6-month delay in care? Why yes/no? Or: in which patient group do you think this is (not) the case? 4. Do you expect patients to switch healthcare providers or seek a second opinion as a result, despite the advice to reschedule the consultation? 5. Is this the same for every patient population or do you think there are differences? Please explain. |
| **Ending** |
| Is there anything else we haven't discussed but is important to mention regarding the topic? Give brief summary of the interview. Thank the expert. |
