# Supplementary material for: Remote Eye Triage: Health Economic Perspectives on Resource Prioritization
Source: Health Serv Insights. 2025 Jun 26;18:11786329251347684. doi: 10.1177/11786329251347684 (PMC12202912; doi:10.1177/11786329251347684)
Supplement: sj-docx-2-his-10.1177_11786329251347684 – Supplemental material for Remote Eye Triage: Health Economic Perspectives on Resource Prioritization [file sj-docx-2-his-10.1177_11786329251347684.docx]

**Supplementary File 2: Complex Interventions Framework**

**Core element 1: context:** Context is important since a complex intervention can be effective in one setting, yet ineffective or even harmful in others.^38^ Here, the context is specified as within outpatient clinic of the Ophthalmology Department of the University Medical Center Utrecht (UMCU) and was developed to cope with sudden downscaling (-90%) due to the covid pandemic. Its initial goal was to identify the most urgent and severe cases. In the post-covid era TTT remained active using the same way of working, yet shifted more towards optimizing resource allocation and critically appraising the need for academic care.

**Core element 2: purposeful program theory:** We used the purposeful program theory by Funnel et al. to develop a pipeline LOGIC model that visualizes the input, processes, output, and outcomes of the potential patient pathways, see figure 1.^8,39,40^ Visualizing the pathways via a LOGIC model simplifies understanding the sequential steps of this complex intervention, thereby making it easier to identify effects, outcomes, or obstacles.

**Core element 3: uncertainties:** Key uncertainties are assessed using the purposeful program theory by Funnel et al. To identifyinfluencing factors which (unintended) affect the expected outcomes, described in Figure 1. Exploring influencing factors can identify factors that should be optimized to be able to realize the mapped potential outcomes. Moreover, this exploration gives the opportunity to inform intervention optimilisation and future data collection on quantitative process evaluation. In the case of eye triage, it could evaluate whether the TTT is expected to behave different in another setting (e.g. in different populations, clinics, or types of care)

**Core element 4: economic considerations:** Fourth, Skivington the Medical Research Coucil encourages to economically monitoring during the whole intervention.^5,41^

Due to the complexity of the intervention during COVID, a conventional cost-benefit or cost-consequence analysis was not feasible. Therefore, a room for improvement analysis is used analyzing theoretical societal costs and QALY’s during a literature review. This process is elaborated below (‘Section Step 3’).

**Supplementary file 3: Assumptions for cost calculations per key diagnosis**

**General comments**

- Utilities were preferebly extrapolated from the most recent European studies. If not, we first looked at the most comparable health care systems, then the United States, then to other regions.
- We expected informal and productivity costs to only occurs when visual acuity quickly restored after treatment. If not, there would be no difference in outcome due to the TTT.

**Cataract**

- Does not include medical saved cost due to delay. Surgical costs are not included, since it is assumed that the surgery itself would take place anyway, though a few months later.
- No difference in consultations is expected, since after delay, a surgical intake consult still needs to take place.
- Since patients have longer burden of VA decrease, informal care and productivity costs are included fully.
- No missed events are expected to occur becaue the likelihood of significant events is exceedingly low.

**Age-related macular disease (AMD)**

- Patients were assumed to at least have (had) wet AMD and treatment with intra-ocular injections, since they are treated in an academic clinic.
- Includes direct medical costs saved due to delay of care such as saved consults and medication and additional costs due to informal care, producitivty or missed events.
- Only patients that have long term stable symptoms are included for the TTT with injection schedules >12 weeks (on which is expected by specialists that these are eligible for longer schedules).
- In case of delay, only a difference in outcomes is expected when a missed event occurs. If no events occur, the patient is not expected to have additional health care consumption, extra costs, of QALY differences. Therefore, for this diagnosis, only the effects on missed events are included.
- A missed events is defined as missed choroidal neovascularization (CNV), which results in other ocular conditions for which treatment with intravitreal injections are required (IVI). For a missed event, the costs of consultation, scans, and medications are included.

**Diabetic retinopathy (DRP)**

- Includes direct medical costs saved due to delay of care, e.g. saved consults and medication.
- Additional costs due to informal care or productivity costs are not included, since the delayed patient without missed events is not expected to be different compared to the non-delayed patient.
- Patients were assumed to at least have background retinopathy, since they are treated in an academic clinic.
- A missed event in interpreted as diabetic macular oedema.

**Glaucoma**

- Includes direct medical costs saved due to delay, e.g. saved consults and medication due to delay
- Additional costs due to informal care or productivity costs are not included, since the delayed patient without missed events is not expected to be different compared to the non-delayed patient.
- Patients were assumed to at least have mild glaucoma, since they are treated in an academic clinic.
- A medical event is interpreted as ‘acute glaucoma’.

**Dry eye syndrome (DES)**

- Patients were expected to have at least mild to moderate DES, since they are treated in an academic clinic.
- Includes direct medical costs saved due to delay of care, e.g. saved consults and medication.
- Since patients have longer burden of VA decrease, productivity costs are included fully.
- Informal care is not expected, since this disease is not expected to harm someone’s life in a way they need informal care, contracy to the diagnoses above.
- No additional surgery costs or punctal plugs are assumed, since it is assumed that the surgery is independent from a missed event, and therefore would take place without the TTT as well.

**Supplementary file 4: keywords and search criteria**

The keywords for the searches were besides the diagnosis, ‘’cost*’’, ‘’societal costs”, ‘’effectiveness’’, ‘’utility’’, ‘’quality-adjusted life year*’’, ‘’screening programs’’, “HTA”, ‘’interventions’’, ‘’delayed treatment’’, and “cost-of illness”. Included studies were: randomized controlled trials, prospective-, retrospective- and observational trials, systematic reviews, and model-based cost-effectiveness studies, all in adults diagnosed with one of the key diagnoses.

**Supplementary file 5: Characteristics expert interviews**

| Characteristics of the interviewees. | |
| --- | --- |
| **Interviewees** | **Variables** |
| Participants | n=5 |
| Nationality | Dutch (n=5) |
| Expertise in Ophthalmology | Cornea and refractive surgery (n=3)  Cataract (n=3)  Retina (n=1)  General Ophthalmology (n=2) |
| Expertise in supervising TTT | Yes (n=3) |
| Expertise in telemedicine | Yes (n=2) |
| Hospital setting | Academic (n=5)  Community hospital/ambulatory (n=2) |
| Involved in ophthalmology policy | Yes (n=5) |

| Supplementary Table 1. Background of included studies included from the review for costs and quality of life. | |
| --- | --- |
| **Cataracy surgery** | |
| ECCERT, Japan 2011^24^ | Utility study. Observational prospective design, 11 clinical sites. N=440. Recruitment: 2008-2010. Utility: trained interviewers: TTO, HUI-3, EQ5D3L. Discounted: 3% |
| Brown, US 2019^42^ | Utility study. model analysis in 2018, dataset of 127685 eyes. Utility: TTO from vision decrease, compared with US life expectancy tables. Discounted: 3% |
| **Age-related macular disease (AMD)** | |
| Brown, US, 2000^28^ | Utility study. Cross-sectional survey study. Inclusion year unknown. N=80. Included AMD patients with VA loss of minimum 50% in at least 1 eye. Utility: by interviewers. TTO and SG. No discount. |
| Neubauer, Germany, 2012^27^ | Cost-effectiveness 10-year Markov model analysis ranibizumab with best supportive care. Clinical data via the ANCHOR and MARINA study. Discounted: 3%. Health insurance perspective. |
| **Diabetic retionopathy (DRP)** | |
| Lloyd, UK, 2008 ^20^ | Utility study. Interview study from five clinical sites, N=122. Utility: by interviewers, SG, EQ5D, HUI3. Year of inclusion unknown. Discounted 3.5% |
| Heintz, Sweden, 2012^32^ | Cost of illness, population-based register study in 2008, N=12.026. Included direct medical costs. No discount. |
| **Glaucoma** | |
| Kobelt, Sweden, 2006^30^ | Utility study. Cross-sectional from four clinics, n=199. Year of inclusion unknown. Utility: self-administered EQ-5D-3L. No discounting. |
| Hagman, Finland, 2013^29^ | Retrospective costing study over 11 years, 2 clinical sites (n=168). 1999 – 2012. Only direct medical costs. No discount, costs recalculated to 2006. |
| **Dry Eye Syndrome (DES)** | |
| Schiffman, US, 2003 ^22^ | Utility study. Observational and prospective design in one clinical site (n=43). Inclusion in 2000-2001. Utility: self-administered TTO. No discount. |
| Mizuno, Japan, 2012^21^ | Costing study. Observational prospective cohort design, 15 clinical sites. (n=118) Inclusion 2005 – 2008 Annual direct medical costs. No discount, calculated to 2008. |
| **General data articles** | |
| Köberlein, 2013 ^18^ | Systematic review in 2012 according to the PRISMA guidelines assessing the international and European economic burden of visual impairment. Included 22 studies. Main contributors were informal care and productivity. |
| Marques, 2021 ^17^ | Model study from 2021 as part of the *Lancet Global Health Commission on Global Eye Health* using economic, demographic and prevalence data (2018-2020) to calculate the global economic productivity losses due to vision impairment and blindness worldwilde, but also European specific. |
| ZorgInstituut Nederland, the Netherlands, 2024 ^35^ | Dutch pricing guideline from 2023 of medical reference prices in health care. |
| CBS, the Netherlands, 2021 ^37^ | Dutch Central Statistics Office (Centraal Bureau voor Statistiek; CBS) which collects data on various social and economic themes in the Dutch society, amongst others labor background. This data is processed into statistical information. |
| Borger, the Netherlands, 2003 ^26^ | Population-based prospective cohort study in 2003 (N=6339) from the Rotterdam Study for whom complete information on prevalence of eye disease status was present. |
| Viso, Spain, 2009 ^23^ | Prevalence study on dry eye disease. Administered questionnaires in 2005-2006 (N=654) |
| Thomas, 2015 ^33^ | Cross-sectional analysis of the National Diabetic Retinopathy Screening Service in Wales in 2005-2009 (N=91.393) to determine prevalence and risk factors. |
| US=United States. UK=United Kingdom. TTO=Time Tradeoff, SG=Standard Gamble. EQ5D=EuroQol-5-dimensions HUI3=Health Utility Index-3. | |
